# Macrophage‐Marker Response and Exploratory Hepatorenal Safety of a Tricalcium Silicate–Biogenic Calcium Carbonate (*Achatina fulica*) Hybrid Pulp‐Capping Material in a Mechanically Exposed Rat Molar Pulp Model: An In Vivo Study

**DOI:** 10.1155/ijod/8074092

**Published:** 2026-10-11

**Authors:** Endo Sadewa, Nurhayaty Natsir, Nurlindah Hamrun, Noor Hikmah, Nisrina Ekayani Nasrun, Kezia Rachellea Mustakim, Juni Jekti Nugroho

**Affiliations:** ^1^ Department of Conservative Dentistry Faculty of Dentistry Hasanuddin University Makassar Indonesia unhas.ac.id; ^2^ Department of Oral Biology Faculty of Dentistry Hasanuddin University Makassar Indonesia unhas.ac.id; ^3^ Department of Oral and Maxillofacial Surgery Faculty of Dentistry Hasanuddin University Makassar Indonesia unhas.ac.id

**Keywords:** *Achatina fulica*, animal model, biocompatibility, calcium carbonate, dental pulp capping, macrophage polarization, tricalcium silicate

## Abstract

**Objectives:**

This study aimed to evaluate, in vivo, the macrophage marker response and exploratory hepatorenal safety of a complete candidate formulation of 75% tricalcium silicate (TCS) and 25% biogenic calcium carbonate (CaCO_3_) from *Achatina fulica* shells, applied to mechanically exposed rat molar pulp, and compared with the manufacturer‐prepared commercial calcium silicate standard.

**Materials and Methods:**

Forty‐eight male Wistar rats underwent mechanical pulp exposure of the maxillary first molars and were randomly allocated to three groups (*n* = 16): negative control (saline), positive control (undiluted commercial calcium silicate cement, manufacturer‐hydrated, designated “100% TCS” for brevity), or the water‐activated hybrid (75% TCS + 25% *A. fulica* CaCO_3_), with euthanasia on days 1, 3, 7, and 14 (*n* = 4/subgroup). Characterization was performed using Fourier‐transform infrared spectroscopy (FTIR) and scanning electron microscopy with energy‐dispersive X‐ray spectroscopy (SEM‐EDX). The pulpal response was assessed by immunohistochemical cell counts for CD68 (pan‐macrophage), inducible nitric oxide synthase (iNOS) (M1‐associated), and CD163 (M2‐associated), and systemic safety by hepatorenal biochemistry (AST, ALT, BUN, creatinine) and blinded liver/kidney histopathology. The prespecified significance threshold was set at *α* = 0.05. Primary inference was drawn from between‐group comparisons pooled across the four time points (*n* = 16 per group), with time‐point‐specific comparisons (*n* = 4) reported descriptively.

**Results:**

FTIR confirmed the presence of characteristic carbonate functional groups, and EDX indicated a calcium fraction of 23.7% on the analyzed surfaces, while chlorine decreased from 13.9% to 4.7% after hydration‐liquid substitution. Compared to the commercial standard, the hybrid was associated with lower pan‐macrophage infiltration (CD68) and M1‐associated marker (iNOS) counts at each timepoint and higher M2‐associated marker (CD163) counts, peaking at day 14 (all omnibus comparisons *p* < 0.001). Hepatorenal parameters and organ histopathology in the hybrid group were milder than those in the commercial standard group, although all four biochemical parameters remained above those of the operated saline control group, and hepatic portal inflammation was modestly higher in the hybrid group (*p* = 0.009).

**Conclusions:**

Compared with the commercial standard, the water‐activated 75:25 hybrid was associated with lower pro‐inflammatory (M1‐associated) and higher proresolving (M2‐associated) macrophage‐marker levels and with milder renal histopathology and minimal hepatic injury relative to the commercial standard. Because these associations reflect marker immunoreactivity in independent cross‐sectional samples rather than tracking individual cells, they support further preclinical evaluation of the hybrid for vital pulp therapy rather than proving efficacy. Because the formulations differed in more than one respect, the results reflect the net effect of the whole formulation rather than the isolated contribution of calcium carbonate.

## 1. Introduction

Encased in rigid mineralized dentin, the dental pulp is a densely vascularized and innervated connective tissue [1]. This casing protects the pulp mechanically, but it comes at a cost: when acute inflammation drives vascular edema, the chamber cannot expand to accommodate it [2]. Any insult to the dentin–pulp complex, such as deep caries, thermal or mechanical injury, or a chemical irritant, provokes an innate immune response that is both rapid and acutely sensitive to intrapulpal pressure [3].

Odontoblasts lining the dentin–pulp interface are usually the first cells to register such an insult [4]. Best known for forming dentin, they also double as nonprofessional immune sentinels, displaying pattern‐recognition receptors that include Toll‐like receptors TLR2 and TLR4 alongside NOD‐like receptors [1, 5, 6]. Once these receptors detect damage‐ or pathogen‐associated molecular patterns, MyD88‐dependent signaling is engaged, and NF‐κB translocates to the nucleus [3, 7, 8]. The cell then releases pro‐inflammatory mediators, IL‐1β, TNF‐α, and IL‐6, which establish a chemotactic gradient and draw circulating leukocytes into the injured tissue [4, 9].

Whether this reaction resolves or tips over into tissue destruction is largely determined by macrophages [10]. Their overall density is conveniently tracked using the pan‐macrophage marker CD68, a lysosomal glycoprotein [11], whereas the surrounding cytokine environment governs their polarization [12]. In the earliest phase, classically activated (M1) macrophages, commonly identified by inducible nitric oxide synthase (iNOS), predominate [10, 13, 14]. M1 activity is indispensable for antimicrobial defense; however, when it persists, the nitric oxide and reactive oxygen species generated begin to injure resident pulp cells and their progenitors [2, 12, 15, 16]. Therefore, healing hinges on a well‐timed hand‐off to the reparative, alternatively activated (M2) phenotype, which carries the scavenger receptor CD163 [13, 17–19]. M2 macrophages quiet inflammation, clear apoptotic debris, drive angiogenesis, and steer pulp stem cells toward odontogenic differentiation [6, 7]—events that culminate in a reparative dentin bridge [20–22]. In broad terms, the balance between iNOS‐associated and CD163‐associated macrophage populations tracks the trajectory of pulpal repair [17, 23, 24]. It should be emphasized that neither marker is phenotype‐exclusive: iNOS is expressed by cell types other than M1 macrophages, CD163 is M2‐associated rather than M2‐specific, and the M1/M2 dichotomy is a simplification of a continuum of macrophage states.

Vital pulp therapy relies on bioactive capping materials to seal the exposure, reduce inflammation, and encourage hard tissue formation [25]. The present benchmark is tricalcium silicate (TCS; Ca_3_SiO_5_)‐based cement, which surpasses calcium hydroxide in terms of biocompatibility, sealing ability, and bioactivity [26, 27]. Upon hydration, it forms a calcium silicate hydrate (C‐S‐H) gel that steadily releases calcium and hydroxyl ions [15]. However, this strength is also a liability for this model. Freshly mixed TCS dissolves quickly and drives a sharp alkaline spike (pH >12.5) at the tissue interface [28, 29]. The resulting alkaline and osmotic stress can produce superficial coagulation necrosis and cause significant loss of pulp cell viability in vitro [28, 30, 31]. These secondary damage signals may prolong M1‐associated marker expression and postpone the shift toward M2‐associated marker expression [32]. Meanwhile, a cement that sets slowly may leach unbound elements into circulation, with potential repercussions for hepatic and renal markers [29, 33].

Blending with calcium carbonate (CaCO_3_) has been proposed as a method to blunt the initial alkalinity while speeding up matrix stabilization [25]. Working through the carbonate–bicarbonate buffer, CaCO_3_ takes up excess hydroxyl ions and may pull the local pH back toward a gentler range without abolishing the antibacterial activity [30, 33]. The particles may serve a second purpose, acting as heterogeneous nucleation sites during hydration [34, 35]. One practical and sustainable reservoir of biogenic CaCO_3_ is the shell of the giant African land snail, *Achatina fulica* [32, 36, 37]. When milled and heat‐treated, they yield calcium‐rich calcite micro‐ and nanoparticles [38, 39]. Early studies have reported good cytocompatibility and the induction of regeneration‐associated growth factors [40]. Reports of low heavy metal content in such preparations are based on techniques other than those used here and were not verified in the present study.

However, longitudinal in vivo evidence is still lacking. No study has yet followed, over time, how an *A. fulica* CaCO_3_/TCS hybrid reshapes macrophage‐marker dynamics (CD68, iNOS, and CD163) in the mechanically exposed pulp, nor has it documented the systemic hepatorenal profile of such a material once it is placed within the tooth [40]. Therefore, we framed a pragmatic, translational comparison—the complete candidate formulation, a 75:25 TCS/biogenic CaCO_3_ hybrid mixed with deionized water, set against the commercial calcium silicate standard prepared to the manufacturer’s specification—rather than an attempt to isolate the independent contribution of calcium carbonate. Our working hypothesis was that, next to the commercial standard, the hybrid would be associated with lower pan‐macrophage infiltration, lower iNOS‐positive cell counts, and higher CD163‐positive cell counts [23, 41] and with hepatic and renal values closer to those of the operated saline control [30, 32].

## 2. Materials and Methods

### 2.1. Ethical Approval, Animals, and Eligibility

This study was approved by the Institutional Health Research Ethics Committee of the Faculty of Dentistry, Hasanuddin University (approval no. 347/KEPK FKG‐RSGMP UH/EA/XII/2025) and conducted in accordance with the ARRIVE 2.0 guidelines [42] and the institutional animal research regulations. The reporting also followed the PRIASE 2021 recommendations for animal studies in endodontology [43]. Forty‐eight male Wistar rats (*Rattus norvegicus*), aged 12–16 weeks and weighing 300–350 g, were used to ensure occlusal maturity. Animals were acclimatized for 7 days under specific‐pathogen‐free (SPF) conditions (22 ± 2°C, 50%–60% relative humidity, and 12‐h light/dark cycle) and group‐housed (two to three per cage) with environmental enrichment. A standard chow diet and filtered water were provided ad libitum. The inclusion criteria were pure‐strain male Wistar rats of the specified age and weight, housed under SPF conditions, and with intact molar anatomy. Animals were excluded if they had weight loss exceeding 10% during acclimatization, pre‐existing dental anomalies, or respiratory complications during anesthesia. No animal died before scheduled euthanasia, met an exclusion criterion after allocation, or was excluded from the analysis.

The experimental unit was an individual animal in each group. Both maxillary first molars of each rat received the same assigned material, and the readings from the two teeth and multiple fields within each section were averaged to yield a single value per animal for every outcome; therefore, teeth, sections, and fields were never treated as independent observations. The resulting animal‐level values for every outcome are provided in File S1, and the script that reproduces every reported statistic from these values is provided in File S2.

The sample size was determined a priori in G ^∗^Power (v3.1.9.7) for a three‐group one‐way analysis of variance, assuming a large effect size (Cohen’s *f* = 0.85), *α* = 0.05, and power (1−*β*) = 0.80. Rederivation from these parameters indicated that this specification required six animals per group (total *N* = 18) for a single three‐group comparison; the present design allocated four animals per group at each of the four time points (16 per group and 48 in total). Consequently, the pooled between‐group comparison (*n* = 16 per group) was well powered under the assumed effect size, whereas any single time‐point three‐group comparison (*n* = 4 per group) attained only ~60% power and could detect only very large effects (*f* ≈1.07 for 80% power). The assumed effect size was not derived from the pilot data. The allocation of four animals per time point was chosen to satisfy the 3R principles while retaining feasibility; the resulting limitations on precision are stated explicitly in Section 4.1, and time‐point‐specific comparisons are reported descriptively rather than as confirmatory inference.

### 2.2. Biogenic Calcium Carbonate Synthesis

CaCO_3_ was extracted from *A. fulica* shells using thermomechanochemical precipitation. The cleaned shells were washed, dried at 110–115°C for 24 h, milled, and sieved through a 100‐mesh screen. The powder was calcined in a muffle furnace at 900°C for 5 h to convert the carbonate into reactive calcium oxide (CaO). For purification, 50 g of CaO was suspended in 2 M nitric acid and stirred (30 rpm) at 60°C for 30 min. Ammonium hydroxide was titrated to pH 12, followed by filtration (Whatman No. 42). Carbon dioxide was then bubbled through the filtrate to precipitate CaCO_3_, which was washed with deionized water to achieve neutrality and vacuum‐dried at 110°C for 2 h, yielding a micrometric CaCO_3_ powder.

### 2.3. Biomaterial Preparation and Characterization

The commercial calcium silicate cement Biodentine (Septodont, Saint‐Maur‐des‐Fossés, France; box lot B34261, powder capsule lot B33530, liquid lot B32671; expiry September 2026) was used as the base material. According to the manufacturer, its powder comprises TCS as the principal constituent, together with dicalcium silicate, calcium carbonate, zirconium oxide (radiopacifier), and iron oxide (colorant), while its liquid contains calcium chloride (setting accelerator) and a water‐soluble polymer [44]. The undiluted commercial cement is designated “100% TCS” for brevity; it is not a chemically pure TCS.

Three formulations were prepared: (i) pure *A. fulica* CaCO_3_; (ii) undiluted commercial cement, hydrated with the manufacturer’s liquid; and (iii) a hybrid comprising 75% commercial powder and 25% *A. fulica* CaCO_3_ by mass, activated with deionized water. The commercial cement was proportioned according to the manufacturer’s protocol at 0.7 g powder to 0.18 mL liquid (powder‐to‐liquid ratio ≈3.9 g mL^−1^) and hand‐mixed on a mixing pad with a stainless‐steel spatula for 30–45 s, following the manufacturer’s manual‐mixing method [44]. For the hybrid, 0.175 g of *A. fulica* CaCO_3_ replaced an equal mass of commercial powder (0.525 g cement + 0.175 g CaCO_3_ = 0.7 g total). The two powders were geometrically blended to homogeneity and activated with deionized water at the same powder‐to‐liquid ratio (0.18 mL per 0.7 g) and blended on a mixing pad with a spatula for 30–45 s. A fresh mixture was prepared for each application at an ambient laboratory temperature (23 ± 2°C). The manufacturer‐specified working time was up to 6 min, and the final setting time of the commercial cement was 10–12 min. The phrase “cross‐activated with pure deionized water” used in the original submission has been replaced with this procedural description.

Because the commercial powder already contains calcium carbonate and zirconium oxide, and because the hybrid was activated with deionized water rather than the manufacturer’s calcium‐chloride‐containing liquid, the two active groups differed simultaneously in three respects: (i) the incorporation of 25% biogenic calcium carbonate, (ii) the substitution of the manufacturer’s calcium‐chloride‐containing hydration liquid with deionized water, which also removes calcium‐chloride exposure, and (iii) the corresponding 25% reduction in the commercial‐powder fraction. This confound is central to the interpretation of the study and is addressed explicitly in Section 4.1.

The functional groups in the shell extract were analyzed by Fourier‐transform infrared spectroscopy (FTIR) in the transmittance mode over 400–4000 cm^−1^ on a Shimadzu spectrophotometer. The peak positions were determined using the instrument’s automated peak‐picking routine, which reports the transmittance minimum, baseline‐corrected intensity, baseline limits used, and integrated and baseline‐corrected areas for each band. The complete instrument peak table for the 13 bands is provided in File S3. A single spectrum was acquired; replicate spectra were not obtained, and the instrument model, sampling mode, spectral resolution, and number of coadded scans were not recorded (Section 4.1). The surface morphology, particle size, and elemental composition were assessed using scanning electron microscopy with energy‐dispersive X‐ray (SEM‐EDX) (TESCAN VEGA) on Au/Pd‐sputter‐coated specimens at an accelerating voltage of 20 keV and magnifications of 500×, 3000×, and 7480×. Elemental quantification was derived from a single map‐sum spectrum for each formulation. The reported uncertainties are the instrument’s spectral fitting standard deviations and are therefore not estimates of between‐specimen or between‐region variability. Au and Pd originating from the conductive coating were excluded from the quantification. The pH and initial setting time of the set hybrid were determined in vitro and are reported in Section 3.1. Neither determination followed a standardized protocol (Gilmore or Vicat needle under ISO 6876 or ISO 9917 conditions), the apparatus and immersion conditions were not recorded, and the commercial cement was not measured under the same conditions; these constraints are stated in Section 4.1.

### 2.4. Pulp Exposure, Grouping, and Application

The maxillary first molar of Wistar rats was selected as a translational model because rat molar pulp is anatomically, histologically, and biologically comparable to human molar pulp and is an established model for direct pulp capping research [45]. Standardized cavity preparation with mechanical pulp exposure produces a reproducible acute inflammatory response suitable for studying the modulatory effect of capping materials [46], and immediate capping after exposure follows standard direct‐pulp‐capping protocols [47].

Under general anesthesia induced by intramuscular injection of ketamine hydrochloride (50 mg/kg) combined with xylazine (5 mg/kg) and antisepsis with 10% povidone‐iodine and 70% alcohol, both maxillary first molars were prepared with a 1/4 round diamond bur (Mani, Japan) under sterile saline irrigation, advancing in increments of ~0.5 mm until the pulp chamber roof was penetrated, and pulp exposure was confirmed by localized bleeding. Hemostasis was achieved using sterile saline‐soaked cotton pellets (Figure 1A). Adequate anesthetic depth was confirmed before cavity preparation and was monitored throughout by the loss of the limb withdrawal response to a noxious stimulus, absence of the pupillary reflex, and a deepened respiratory pattern. Preparation began only after all three criteria were met.

**Figure 1:**
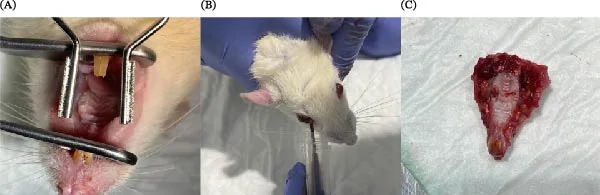
Photographic documentation of the in vivo experimental workflow: (A) operative field with the maxillary first molars exposed under general anesthesia, (B) blood collection for systemic biochemical analysis, and (C) the resected maxillary block prepared for fixation and histological processing.

The 48 animals were randomly allocated to three groups (*n* = 16 each) using a computer‐generated sequence, with allocation concealed in sequentially numbered, opaque, sealed envelopes opened by an assistant not involved in surgery: Group 1 (negative control) received saline only; Group 2 (positive control) received manufacturer‐hydrated commercial cement; and Group 3 (hybrid) received water‐activated 75:25 paste. In the two active groups, a single increment of freshly mixed paste was placed directly onto the exposure site with a sterile applicator and gently adapted to completely cover the exposure site and the surrounding cavity floor. The increment was not weighed, and no mass was claimed. The material was applied in a freshly mixed state and set in situ within the sealed cavity. All sites were sealed with resin‐modified glass‐ionomer cement (GC, Japan) and light‐cured for 40 s. Meloxicam (1 mg/kg, subcutaneously) was administered immediately after surgery and daily for 3 days. Euthanasia was performed on days 1, 3, 7, and 14 (*n* = 4 per subgroup) by inhalation of diethyl ether until deep anesthesia was established over 5–15 min, followed immediately by decapitation. No barbiturates were administered.

### 2.5. Systemic Safety Assessment

Blood (0.8–1.0 mL) was collected under terminal inhalational anesthesia, as described in Section 2.4, immediately before decapitation (Figure 1B). Hepatic (AST and ALT) and renal (BUN and creatinine) markers were measured colorimetrically using a biochemical autoanalyzer. Liver and kidney tissues were excised, fixed in 10% neutral‐buffered formalin, processed routinely, and stained with hematoxylin and eosin.

The sections were scored by an independent pathologist blinded to the group allocation. Kidney sections were graded on a four‐point (0–3) scale for four features: glomerular change, tubular injury, interstitial inflammation, and hemorrhage, using prespecified criteria (0 = normal; 1 = minimal/mild; 2 = moderate; and 3 = severe), with at least 100 cortical tubules assessed per specimen for tubular injury and 10 random high‐power fields assessed for interstitial inflammation and hemorrhage. The four‐feature scores were summed to obtain an aggregate renal injury score (0–12) for each animal. Liver sections were graded on the same 0–3 scale for 13 features grouped as hepatocellular injury (degeneration/vacuolization, microvesicular steatosis, macrovesicular steatosis, necrosis, and apoptosis), inflammation (portal, lobular, and Kupffer‐cell activation), cholestatic/vascular change (sinusoidal congestion/dilatation, hemorrhage, and cholestasis), and chronicity (ductular reaction, fibrosis), assessed over 10 random fields at 100× magnification with an overall survey at 40×. Feature definitions followed the National Toxicology Program Nonneoplastic Lesion Atlas [48], and the 13 scores were summed to obtain an aggregate hepatic score (0–39) for each animal in the study.

### 2.6. Immunohistochemistry

Maxillary blocks (Figure 1C) were fixed in 10% neutral‐buffered formalin, decalcified in formic acid for 21 days, embedded in paraffin, and sectioned to a thickness of 4 µm for histological examination. After heat‐induced epitope retrieval in citrate buffer (pH 6.0), endogenous peroxidase was quenched with 3% H_2_O_2_ for 20 min, and nonspecific protein binding was blocked with 5% fetal bovine serum containing 0.25% Triton X‐100. Sections were incubated for 60 min at room temperature with monoclonal primary antibodies (Abcam, Cambridge, UK): anti‐CD68 (ab125212, 1:200), anti‐iNOS (ab15323, 1:100), or anti‐CD163 (ab182422, 1:500) for 40 min with an HRP‐conjugated anti‐rabbit secondary antibody, developed with 3,3′‐diaminobenzidine for 10 min, counterstained with Mayer’s hematoxylin for 10 min, air‐dried, and mounted in Entellan under a cover glass. All steps were separated by three 5‐min washes in phosphate‐buffered saline at pH 7.4.

Immunoreactivity was quantified as the number of immunopositive cells per high‐power field, and a cell was scored as positive when a distinct brown diaminobenzidine chromogen was present in its cytoplasm. Counts were performed manually at 1000× magnification (oil immersion) in five nonoverlapping fields per section within the coronal pulp stroma subjacent to the exposure site and recorded on a worksheet. Blinding was procedural: before assessment, each coded slide had its identifier masked and was assigned a fresh random number, so the observer could not know which group a section belonged to. No digital image analysis, thresholding, or color deconvolution procedure was used, and the number and spacing of sections examined per tooth and the immunohistochemical staining controls were not recorded (Section 4.1). Field values were averaged within a section, sections within a tooth, and the two teeth within an animal so that a single animal‐level value was entered into the analysis. This metric replaces the “H‐score” terminology used in the original submission, which was applied in error; the reported values were never intensity‐weighted 0–300 histological scores.

### 2.7. Statistical Analysis

Analyses were performed using IBM SPSS Statistics Version 29.0 (IBM Corp., Armonk, NY, USA) and verified independently using Python 3.11 (SciPy 1.x, statsmodels). The verification script is provided in File S2. A single significance threshold of *α* = 0.05 was specified and applied throughout the study.

The primary outcome was the between‐group difference in CD163‐positive cell counts, which is the marker that most directly reflects the proresolving response that the formulation is intended to promote. CD68 and iNOS counts, the four hepatorenal analytes, and organ histopathology scores were the secondary outcomes. An a priori power calculation was performed for a single three‐group comparison and was not adjusted for the number of outcomes examined.

Test selection was data‐driven. Both assumption checks were evaluated on the pooled group distributions because they are the distributions to which the primary test is applied: normality by the Shapiro–Wilk test and homogeneity of variance by Levene’s test, applied to each group’s 16 animal‐level values. When the pooled distributions were approximately normal with homogeneous variances (CD68, iNOS, creatinine, and ALT), one‐way ANOVA with the Tukey HSD post hoc test was used. When normality was violated in any group (CD163, BUN, and AST), the Kruskal–Wallis *H*‐test was used with Mann–Whitney *U* pairwise comparisons and Holm adjustment for the three contrasts. Welch’s ANOVA with the Games–Howell post hoc test was reserved for distributions that were normal but heteroscedastic; no outcome fell into that category once the checks were applied at the pooled level. Shapiro–Wilk tests computed within individual group × timepoint cells (*n* = 4) were not used for test selection because at that size, the test has almost no power to detect departures from normality. Ordinal histopathology scores were compared using the Kruskal–Wallis *H*‐test.

For the comparison of primary interest (hybrid versus commercial standard), an effect estimate with a 95% confidence interval is reported alongside each *p*‐value in the Results: a Welch mean difference where the parametric test was used and a Hodges–Lehmann median difference with a distribution‐free interval where the nonparametric test was used.

Because the four time points comprise independent cross‐sectional samples of four animals each, the primary inference was drawn from the between‐group comparison pooled across time points (*n* = 16 per group), which is presented as the primary analysis in the Section 3. Time‐point‐specific values are reported descriptively as mean ± standard deviation, together with the median (minimum–maximum) in the Results tables. Two features of the design constrain time‐point‐specific inference and are stated here to avoid over‐reading those tables: with four animals per group, the smallest attainable two‐sided exact Mann–Whitney *p*‐value is 0.029 (2/70), so no pairwise nonparametric contrast at a single timepoint can fall below that value however complete the separation; the corresponding three‐group Kruskal–Wallis statistic reaches at most *H* = 9.85, which the *χ* ^2^ approximation renders as *p* ≈ 0.007 (the exact permutation value for complete separation is 1.7 × 10 ^4^). The power at a single time point was ~0.60 under the assumed effect size. Accordingly, no time‐point‐specific *p*‐values were presented as confirmatory evidence.

To assess whether the temporal trajectories, rather than only the overall levels, differed between formulations, the group × timepoint interaction was additionally examined for each marker using aligned rank‐transform factorial analysis [49], a method suited for independent cross‐sectional timepoints, nonnormal distributions, and small cell sizes.

## 3. Results

### 3.1. Physicochemical and Microstructural Characterization

The FTIR spectrum of the synthesized *A. fulica* powder (Figure 2) shows a broad O–H stretching centered at 3433 cm^−1^ (adsorbed water/hydroxyl) and the diagnostic vibrational modes of the carbonate ion: an intense asymmetric *ν*_3_ (CO_3_^2−^) stretch at 1400 cm^−1^, the *ν*_2_ out‐of‐plane bend at 874 cm^−1^, and the *ν*_4_ in‐plane bend at 710 cm^−1^, together with the *ν*_1_ + *ν*_4_ combination band at 1796 cm^−1^ and a further combination band at 2513 cm^−1^. The instrument peak table (File S3) lists 13 bands and resolves the *ν*_4_ region as a single band at 709.83 cm^−1^ rather than the 700/713 cm^−1^ doublet of aragonite, with no band near the 854 cm^−1^ aragonite *ν*_2_ position; together with the *ν*_2_ band at 873.78 cm^−1^, this is consistent with calcite‐dominant CaCO_3_. The *ν*_3_ band was saturated at the transmittance floor of the instrument (transmittance minimum 0.53%), and the peak table returned a negative baseline‐corrected area, confirming that this band could not be treated quantitatively. FTIR band positions identify the carbonate functional groups but cannot establish phase purity by themselves, and no purity claim is made based on this spectrum.

**Figure 2:**
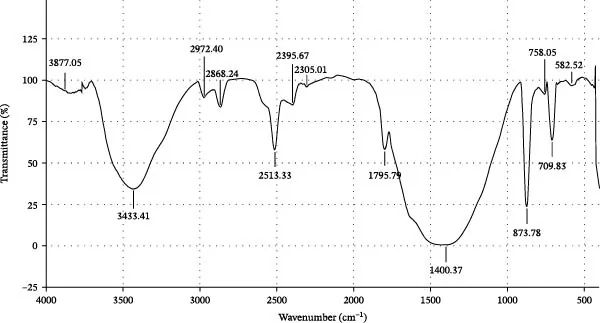
Fourier‐transform infrared (FTIR) transmittance spectrum of *Achatina fulica* shell‐derived calcium carbonate powder. The bands at 3433 cm^−1^ (O–H stretch of adsorbed water), 1796 and 2513 cm^−1^ (carbonate combination bands), 1400 cm^−1^ (*ν*_3_ asymmetric ${\text{CO}}_{3}^{2-}$ stretch), 874 cm^−1^ (*ν*_2_ out‐of‐plane bend), and 710 cm^−1^ (*ν*_4_ in‐plane bend) identify the carbonate functional groups; the *ν*_2_ and *ν*_4_ positions and the absence of an aragonite doublet are consistent with a calcite‐dominant phase. The *ν*_4_ region is resolved as a single band rather than the 700/713 cm^−1^ aragonite doublet, and no band is present near the 854 cm^−1^ aragonite *ν*_2_ position. The *ν*_3_ band is saturated at the transmittance floor, and the band intensities are not interpreted quantitatively. The complete instrument peak table for all 13 detected bands is provided in File S3.

SEM (Figure 3A–C) revealed that calcination removed the residual organic matrix, producing dense crystalline agglomerates. The individual particle diameters measured from these micrographs ranged from ~5.08 to 6.69 µm, a range derived from a small number of manual measurements rather than from a formal particle size distribution. EDX mapping (Figure 3D–H) showed that Ca, oxygen, and carbon were distributed throughout the hybrid matrix in a patchy rather than strictly uniform pattern, consistent with a physical blend of the two particulate phases.

**Figure 3:**
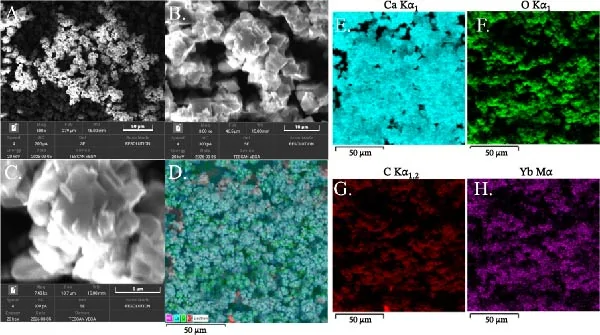
Scanning electron microscopy (SEM) and energy‐dispersive X‐ray (EDX) elemental mapping of the hybrid matrix (TESCAN VEGA, 20 keV) were performed. (A–C) SEM micrographs at 500×, 3000×, and 7480× magnifications showing dense crystalline microparticle agglomeration; the instrument scale bars are shown in each panel. (D) Layered SEM‐EDX map. (E–G) EDX elemental maps of calcium (Ca Kα_1_), oxygen (O Kα_1_), and carbon (C Kα_1,2_) showing a patchy distribution consistent with the physical blend of two particulate phases. (H) Map of the nominal Yb Mα window, retained for transparency only: this signal was flagged as unreliable by the instrument software and interpreted as spectral overlap rather than Yb (see Section 3.1).

Quantitatively (Table 1), chlorine decreased markedly from 13.9 wt% in the commercial cement to 4.7 wt% in the hybrid, consistent with the replacement of the manufacturer’s calcium chloride‐containing liquid with deionized water. Oxygen (40.2 → 47.5 wt%) and carbon (18.2 → 19.8 wt%) increased with carbonate incorporation, while the surface calcium fraction was essentially unchanged (23.4 → 23.7 wt%). Because the shell extract is calcium‐richer than the commercial cement (39.0 versus 23.4 wt%), a simple mass‐weighted expectation would predict a modest increase in surface calcium; the near‐constancy observed is therefore an observation requiring explanation rather than evidence of calcium retention, and with a single spectrum per material, it cannot be distinguished from measurement variation. The minor elements (Si, Al, Mg, and Zr) remained essentially unchanged. The corresponding map‐sum spectra are presented for the pure extract (Figure 4A), the commercial cement (Figure 4B), and the hybrid (Figure 4C). Two caveats apply to Table 1. First, quantification was derived from one map‐sum spectrum per formulation; therefore, no variability across regions or specimens could be reported. EDX is surface‐sensitive and semiquantitative; therefore, these values describe the surface composition and cannot establish bulk stoichiometry, stable calcium retention, ion release, or reduced leaching. Second, the low‐level ytterbium signal returned by the automated quantification routine (0.6 wt% in the shell extract and 0.2 wt% in the hybrid) was flagged as unreliable by the instrument software and is chemically implausible in a biogenic carbonate; the Yb Mα line lies close to the Al Kα and Si Kα region, and this assignment is most plausibly a spectral‐overlap artifact. Accordingly, ytterbium is omitted from Table 1, and no interpretation is placed on it pending confirmation by an element‐specific technique such as ICP‐MS.

**Figure 4:**
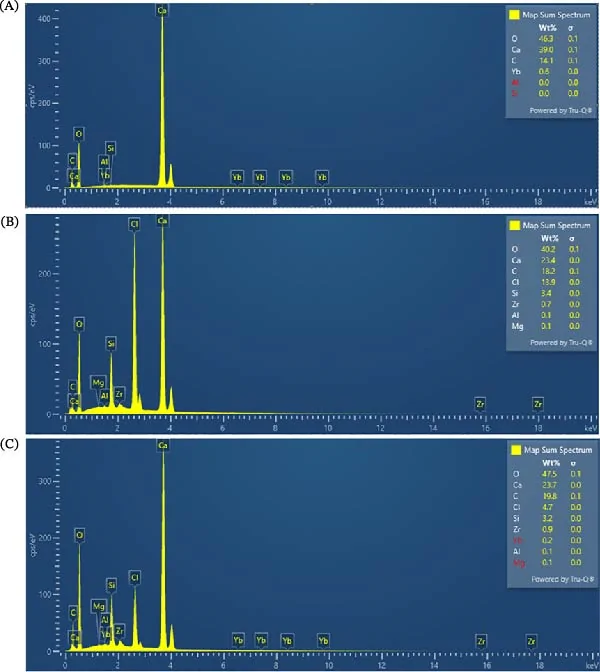
Map‐sum EDX spectra of the three tested formulations: (A) pure *Achatina fulica* calcium carbonate extract, (B) undiluted commercial calcium‐silicate cement, and (C) 75:25 hybrid formulation. One map‐sum spectrum was obtained for each formulation. The tabulated weight percentages and σ values shown in each inset are the instrument’s spectral‐fitting outputs and do not represent the between‐region or between‐specimen variability. The reduction in the chlorine peak in (C) relative to that in (B) corresponds to the substitution of the manufacturer’s calcium‐chloride‐containing liquid with deionized water.

**Table 1:** Surface elemental composition of the tested biomaterials by SEM‐EDX (weight %).

Element	Pure *A. fulica* extract	Commercial cement (“100% TCS”)	Hybrid (75% commercial + 25% *A. fulica*)
Oxygen (O)	46.3	40.2	47.5
Calcium (Ca)	39.0	23.4	23.7
Carbon (C)	14.1	18.2	19.8
Chlorine (Cl)	n.d.	13.9	4.7
Silicon (Si)	n.d.	3.4	3.2
Zirconium (Zr)	n.d.	0.7	0.9
Aluminum (Al)	n.d.	0.1	0.1
Magnesium (Mg)	n.d.	0.1	0.1

*Note:* Values are returned by the instrument’s automated quantification routine and rounded to one decimal place. Au and Pd from the conductive coating were excluded from the quantification. A low‐level ytterbium signal—0.6% in the shell extract and 0.2% in the hybrid—was flagged as unreliable by the instrument software, was chemically implausible in a biogenic carbonate, and most plausibly reflected spectral overlap in the Al Kα/Si Kα region; therefore, it was omitted (see Section 3.1). The extract column consequently totaled 99.4%, rather than 100%, the 0.6% shortfall being precisely the omitted ytterbium; the commercial cement and hybrid columns totaled 100.0%. Values derived from a single map‐sum spectrum per formulation.

Abbreviation: n.d., not detected.

In vitro, the set hybrid exhibited an alkaline pH of 11–12 and an initial setting time of 8–11 min. Three caveats govern the interpretation of these values. First, the measured pH was close to the range expected for a set TCS cement and did not demonstrate pH buffering. Second, the pH of a *set* specimen is not the same as the interfacial pH of freshly mixed cement in contact with pulp fluid, where an alkaline peak above 12.5 occurs, as described in the literature; therefore, the two values are not directly comparable. The pH of commercial cement was not measured in this study, and no within‐study pH comparison between the materials was available. Third, although the hybrid’s initial set at 8–11 min falls at or below the manufacturer‐specified 10–12 min final set of the commercial cement, the initial and final sets are different endpoints determined by different criteria, and the two materials were not measured side by side under a common protocol; therefore, the comparison is only indicative and does not establish accelerated setting.

### 3.2. Systemic Safety: Hepatorenal Biochemistry and Histopathology

The descriptive values for all four hepatorenal markers at each timepoint are given in Tables 2 and 3; the differences were mainly driven by the commercial cement group. Creatinine in that group was approximately three times the saline value at every timepoint (day 1, 48.95 ± 0.68 µmol L^−1^; day 14, 49.55 ± 0.47 µmol L^−1^), whereas the hybrid group, though markedly lower than the commercial standard, remained above the saline control (day 14, 29.73 ± 0.29 µmol L^−1^). The BUN levels followed the same pattern. ALT was elevated with the commercial standard (day 1, 101.00 ± 2.94 U L^−1^) and closer to the baseline with the hybrid (day 14, 81.25 ± 1.26 U L^−1^). The mean AST level in the hybrid group was ~40% lower than that in the commercial cement group (day 1, 256.75 ± 3.50 vs. 423.00 ± 15.43 U L^−1^). Because the values in the commercial cement group changed little between days 1 and 14, these elevations were not described as transient. The temporal profiles with individual animal values are shown for creatinine (Figure 5A), BUN (Figure 5B), ALT (Figure 5C), and AST (Figure 5D). In the pooled between‐group analysis (Table 4), all four markers differed significantly between groups (creatinine and ALT by one‐way ANOVA, BUN and AST by Kruskal–Wallis; all *p* < 0.001), and every pairwise contrast remained significant after adjustment for multiplicity (all *p* < 0.001). Relative to the commercial standard, the hybrid showed lower creatinine (mean difference −19.66 µmol L^−1^, 95% CI −20.02 to −19.30), BUN (Hodges–Lehmann median difference −0.80 mmol L^−1^, 95% CI −0.88 to −0.71), ALT (−20.94 U L^−1^, 95% CI −22.61 to −19.27), and AST (−167.0 U L^−1^, 95% CI −171.0 to −163.0).

**Figure 5:**
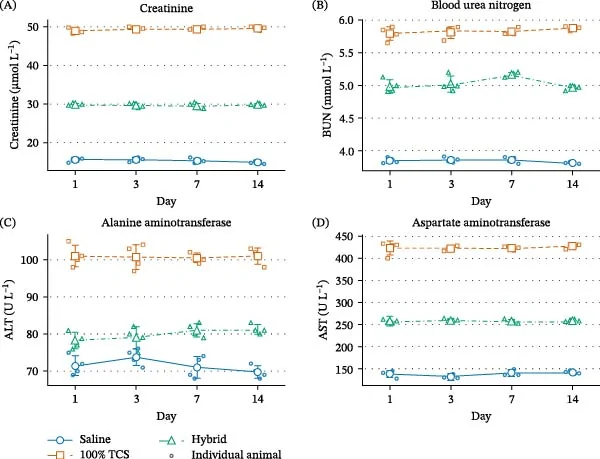
Hepatorenal biochemistry on days 1, 3, 7, and 14: (A) serum creatinine, (B) blood urea nitrogen, (C) alanine aminotransferase, and (D) aspartate aminotransferase levels. Filled symbols and connecting lines show group means with error bars representing ± 1 SD; open symbols show the individual values of all four animals in each subgroup. The values in the commercial cement group were consistently the highest and changed little over time; the hybrid group was intermediate, remaining above the operated saline control for all four analytes.

**Table 2:** Renal biochemical markers were measured on days 1, 3, 7, and 14.

Day	Group	Creatinine (µmol L^−1^)	BUN (mmol L^−1^)
1	Saline	15.53 ± 0.50; 15.7 (14.8–15.9)	3.85 ± 0.04; 3.845 (3.81–3.9)
	100% TCS	48.95 ± 0.68; 48.95 (48.2–49.7)	5.79 ± 0.11; 5.815 (5.65–5.89)
	Hybrid	29.83 ± 0.29; 29.75 (29.6–30.2)	4.98 ± 0.10; 4.955 (4.9–5.12)
3	Saline	15.63 ± 0.51; 15.75 (14.9–16.1)	3.86 ± 0.04; 3.855 (3.82–3.91)
	100% TCS	49.33 ± 0.46; 49.25 (48.9–49.9)	5.81 ± 0.09; 5.825 (5.69–5.89)
	Hybrid	29.60 ± 0.56; 29.7 (28.9–30.1)	5.02 ± 0.13; 4.985 (4.91–5.2)
7	Saline	15.27 ± 0.65; 15.1 (14.7–16.2)	3.86 ± 0.05; 3.865 (3.8–3.9)
	100% TCS	49.38 ± 0.43; 49.35 (48.9–49.9)	5.82 ± 0.05; 5.795 (5.78–5.89)
	Hybrid	29.43 ± 0.75; 29.35 (28.7–30.3)	5.16 ± 0.04; 5.155 (5.12–5.2)
14	Saline	14.85 ± 0.29; 14.85 (14.5–15.2)	3.81 ± 0.01; 3.81 (3.8–3.83)
	100% TCS	49.55 ± 0.47; 49.65 (48.9–50)	5.87 ± 0.03; 5.875 (5.83–5.9)
	Hybrid	29.73 ± 0.29; 29.65 (29.5–30.1)	4.97 ± 0.03; 4.975 (4.92–4.99)

*Note:* Each cell shows the mean ± SD and median (minimum–maximum). *n* = 4 animals per subgroup in each group. Saline = negative control; 100% TCS = positive control (undiluted commercial calcium silicate cement); hybrid = 75% commercial powder + 25% *A. fulica* CaCO_3_, water‐activated.

**Table 3:** Hepatic biochemical markers were measured on days 1, 3, 7, and 14 of the experiment.

Day	Group	ALT (U L^−1^)	AST (U L^−1^)
1	Saline	71.50 ± 2.65; 71 (69–75)	138.00 ± 7.26; 139.5 (128–145)
	100% TCS	101.00 ± 2.94; 100.5 (98–105)	423.00 ± 15.43; 429.5 (400–433)
	Hybrid	78.25 ± 2.22; 78 (76–81)	256.75 ± 3.50; 256.5 (253–261)
3	Saline	73.75 ± 2.22; 74 (71–76)	132.25 ± 4.03; 131 (129–138)
	100% TCS	100.75 ± 3.30; 101 (97–104)	421.50 ± 5.20; 421 (416–428)
	Hybrid	79.00 ± 2.94; 79.5 (75–82)	260.00 ± 2.16; 259.5 (258–263)
7	Saline	71.00 ± 2.94; 71 (68–74)	141.00 ± 6.27; 139.5 (136–149)
	100% TCS	100.50 ± 1.29; 100.5 (99–102)	423.50 ± 3.79; 425 (418–426)
	Hybrid	81.00 ± 1.83; 81 (79–83)	256.75 ± 4.50; 255.5 (253–263)
14	Saline	69.75 ± 1.71; 69.5 (68–72)	142.00 ± 3.16; 141.5 (139–146)
	100% TCS	101.00 ± 2.16; 101.5 (98–103)	427.25 ± 4.99; 429 (420–431)
	Hybrid	81.25 ± 1.26; 81 (80–83)	259.00 ± 2.58; 259 (256–262)

*Note:* Each cell shows the mean ± SD and median (minimum–maximum) values. *n* = 4 animals in each subgroup. The group abbreviations are presented in Table 2.

**Table 4:** Primary inferential analysis: between‐group comparison of each outcome pooled across the four time points (*n* = 16 animals/group).

Outcome	Test	Omnibus *p*	Saline vs. TCS	Saline vs. Hybrid	TCS vs. Hybrid	Effect (95% CI)
CD68	ANOVA	<0.001	<0.001	<0.001	0.023	MD −1.84 (−3.25, −0.44)
iNOS	ANOVA	<0.001	<0.001	<0.001	0.003	MD −1.84 (−3.03, −0.65)
CD163	KW	<0.001	<0.001	<0.001	0.005	HL + 3.00 (+1.00, +5.00)
Creatinine	ANOVA	<0.001	<0.001	<0.001	<0.001	MD −19.66 (−20.02, −19.30)
BUN	KW	<0.001	<0.001	<0.001	<0.001	HL −0.80 (−0.88, −0.71)
ALT	ANOVA	<0.001	<0.001	<0.001	<0.001	MD −20.94 (−22.61, −19.27)
AST	KW	<0.001	<0.001	<0.001	<0.001	HL −167.0 (−171.0, −163.0)

*Note:* ANOVA = one‐way ANOVA with Tukey HSD post hoc; KW = Kruskal–Wallis *H* with Holm‐adjusted Mann–Whitney *U* post hoc; Welch = Welch ANOVA with Games–Howell post hoc (retained in the analysis plan; no outcome required it). MD, Welch’s mean difference; HL, Hodges–Lehmann median difference. Effect estimates are hybrid minus commercial standard, with units as in Tables 2, 3, and 5. Pairwise contrasts were adjusted for three comparisons within each outcome, not across the outcomes. Test selection followed the pooled‐distribution assumption checks: CD68 normal, homoscedastic; iNOS normal, homoscedastic; CD163 nonnormal (Shapiro–Wilk *p* ^∗^ = 0.021); creatinine normal, homoscedastic; BUN nonnormal (Shapiro–Wilk *p* = 0.024); ALT normal, homoscedastic; AST nonnormal (Shapiro–Wilk *p* = 0.011) ^∗^. The group abbreviations are listed in Table 2.  ^∗^ *p* < 0.05 after multiplicity adjustment (Tukey HSD for ANOVA outcomes; Holm correction for Kruskal–Wallis outcomes).

**Table 5:** Immunohistochemical macrophage marker counts (immunopositive cells per high‐power field) were determined on days 1, 3, 7, and 14.

Marker	Day	Saline	100% TCS	Hybrid
CD68	1	11.63 ± 1.25; 11.75 (10–13)	7.50 ± 1.29; 7.5 (6–9)	5.25 ± 0.96; 5.5 (4–6)
	3	13.50 ± 1.29; 13.5 (12–15)	6.75 ± 1.71; 6.5 (5–9)	3.75 ± 0.96; 3.5 (3–5)
	7	11.25 ± 0.96; 11.5 (10–12)	4.63 ± 0.48; 4.75 (4–5)	3.25 ± 1.26; 3 (2–5)
	14	9.25 ± 0.50; 9 (9–10)	2.50 ± 0.58; 2.5 (2–3)	1.75 ± 0.50; 2 (1–2)
iNOS	1	10.00 ± 0.82; 10 (9–11)	6.00 ± 0.82; 6 (5–7)	4.50 ± 1.29; 4.5 (3–6)
	3	9.63 ± 0.48; 9.75 (9–10)	4.75 ± 0.96; 4.5 (4–6)	2.75 ± 0.96; 2.5 (2–4)
	7	9.00 ± 0.82; 9 (8–10)	3.88 ± 1.75; 3.75 (2–6)	2.00 ± 0.82; 2 (1–3)
	14	7.50 ± 0.58; 7.5 (7–8)	3.00 ± 1.83; 3 (1–5)	1.00 ± 0.82; 1 (0–2)
CD163	1	2.63 ± 0.48; 2.75 (2–3)	4.00 ± 0.82; 4 (3–5)	5.50 ± 1.29; 5.5 (4–7)
	3	2.38 ± 0.48; 2.25 (2–3)	6.25 ± 1.26; 6 (5–8)	8.75 ± 1.26; 9 (7–10)
	7	4.00 ± 0.82; 4 (3–5)	7.50 ± 1.29; 7.5 (6–9)	10.50 ± 1.29; 10.5 (9–12)
	14	3.25 ± 1.50; 3 (2–5)	8.25 ± 0.96; 8.5 (7–9)	12.75 ± 1.26; 13 (11–14)

*Note:* Each cell shows the mean ± SD and median (minimum–maximum) values. *n* = 4 animals per subgroup in each group. Group abbreviations are listed in Table 2.

On blinded semiquantitative scoring (Table 6), the aggregate renal injury score mirrored the biochemistry, being lowest with saline (median 1.0), highest with the commercial standard (6.0), and intermediate with the hybrid (3.5; Kruskal–Wallis *p* < 0.001); the glomerular, tubular, interstitial‐inflammatory, and hemorrhagic components differed significantly between groups (all *p* < 0.001). Therefore, the hybrid group showed mild renal changes, most often tubular injury and interstitial hemorrhage, with fewer glomerular and inflammatory changes that were less severe than those seen with the commercial standard but still greater than those in the operated saline controls.

**Table 6:** Blinded semiquantitative histopathology of the kidney and liver, pooled across days 1–14 (*n* = 16 animals/group).

Feature (score 0–3)	Saline	100% TCS	Hybrid	*p*
Kidney				
Glomerular change	0 (0–1)	2 (1–3)	1 (0–1)	<0.001
Tubular injury	0 (0–1)	1.5 (1–3)	1 (0–2)	<0.001
Interstitial inflammation	0 (0–1)	1.5 (1–3)	0.5 (0–2)	<0.001
Hemorrhage	0 (0–1)	1 (0–3)	1 (0–2)	<0.001
Aggregate renal score (0–12)	**1 (0–2)**	**6 (3–12)**	**3.5 (0–7)**	**<0.001**
Liver				
Hepatocyte degeneration	0 (0–3)	0 (0–1)	0 (0–2)	0.581
Microvesicular steatosis	0 (0–1)	0 (0–1)	0 (0–2)	0.458
Necrosis	0 (0–2)	0 (0–0)	0 (0–1)	0.600
Apoptosis	0 (0–1)	0 (0–0)	0 (0–0)	0.368
Portal inflammation	0 (0–1)	0 (0–1)	1 (0–2)	0.009
Lobular inflammation	0 (0–0)	0 (0–0)	0 (0–1)	0.130
Kupffer‐cell activation	0 (0–0)	0 (0–0)	0 (0–1)	0.130
Sinusoidal congestion	0 (0–0)	0 (0–2)	0 (0–1)	0.016
Hemorrhage	0 (0–0)	0 (0–2)	0 (0–1)	0.005
Macrosteatosis, cholestasis, ductular reaction, fibrosis	0 (0–0)	0 (0–0)	0 (0–0)	—
Aggregate hepatic score (0–39)	**0 (0–6)**	**1.5 (0–5)**	**1 (0–7)**	**0.048**

*Note:* Bold values indicate statistically significant differences among the three groups (Kruskal–Wallis H‐test, *p* < 0.05). “—” = no events recorded in any animal; therefore, no test was performed. The aggregate hepatic result was marginal and not robust to alternative handling of the source records (see Section 4.1). Each feature was scored 0–3; values are median (minimum–maximum). Group abbreviations are listed in Table 2. Between‐group comparisons were performed using the Kruskal–Wallis *H*‐test.

Hepatic injury was minimal in all three groups; no macrovesicular steatosis, cholestasis, ductular reaction, or fibrosis was observed in any animal, and hepatocyte degeneration, microvesicular steatosis, necrosis, and apoptosis did not differ significantly among the groups. However, these three features differed. Portal inflammation was modestly higher in the hybrid group than in the control group (median, 1.0 vs. 0; *p* = 0.009), whereas sinusoidal congestion (*p* = 0.016) and hepatic hemorrhage (*p* = 0.005) were more frequent in the two intervention groups than in the control group. The aggregate hepatic score differed only marginally between the groups (medians 0.0, 1.5, and 1.0 for saline, commercial standard, and hybrid, respectively; *p* = 0.048). As noted in Section 4.1, this single borderline result is not robust to plausible alternative handling of the source scoring data. Representative hepatic and renal photomicrographs are shown in Figure 6.

**Figure 6:**
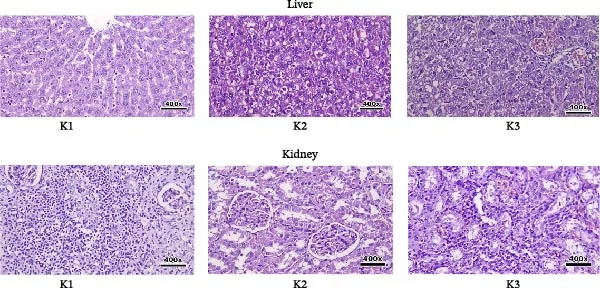
Representative hematoxylin and eosin photomicrographs of hepatic (upper row) and renal (lower row) tissues on day 14, one panel per experimental group for each organ (400× magnification). The hepatic architecture was preserved in all groups, with no macrovesicular steatosis, cholestasis, ductular reaction, or fibrosis observed in any animal. Renal panels illustrated glomerular, tubular, interstitial‐inflammatory, and hemorrhagic changes (Table 6). Panels are labeled K1–K3 in each organ and correspond to Groups 1–3 as defined in Section 2.4: K1, saline; K2, 100% TCS; and K3, hybrid TCS. These images are illustrative only; all quantitative comparisons are presented in Table 6. The original magnification is indicated on each panel (400×); calibrated scale bars are not available for archived micrographs (Section 4.1).

### 3.3. Pulpal Macrophage Markers (CD68, iNOS, and CD163)

Pooled across the four timepoints, all three macrophage markers differed significantly between the three groups, and—the comparison of primary interest—the hybrid differed significantly from the commercial standard for each marker after adjustment for multiplicity: CD68 lower by 1.84 cells per field (95% CI 0.44–3.25; Tukey *p* = 0.023), iNOS lower by 1.84 (95% CI 0.65–3.03; *p* = 0.003), and CD163 higher by a Hodges–Lehmann median difference of 3.00 (95% CI 1.00–5.00; Holm‐adjusted *p* = 0.005) (Table 4). Both active materials differed from the saline control for all three markers (all *p* < 0.001). The time‐point‐specific values are listed in Table 5, representative micrographs are shown in Figure 7, and temporal profiles with individual animal values are shown in Figure 8.

**Figure 7:**
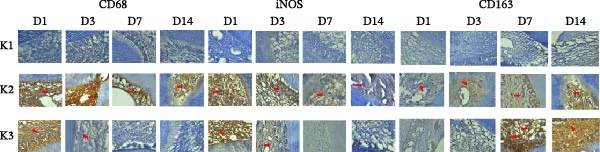
Representative immunohistochemical photomicrographs of CD68, iNOS, and CD163 immunoreactivity in the coronal pulp stroma on days 1, 3, 7, and 14 (1000× magnification, oil immersion). Brown 3,3′‐diaminobenzidine chromogen in the cytoplasm indicates positive immunoreactivity; red arrows indicate immunopositive cells; and the sections were counterstained with Mayer’s hematoxylin. Each block of four columns corresponds to one marker at four time points: The rows are labeled K1–K3 and correspond to Groups 1–3 as defined in Section 2.4: K1, saline; K2, 100% TCS; and K3, hybrid TCS. These images are illustrative of the staining appearance only and are not the basis for any quantitative comparisons. All counts are given in Table 5 and Figure 8, and the counts, not the illustrated fields, support every statement in the Section 3.

**Figure 8:**
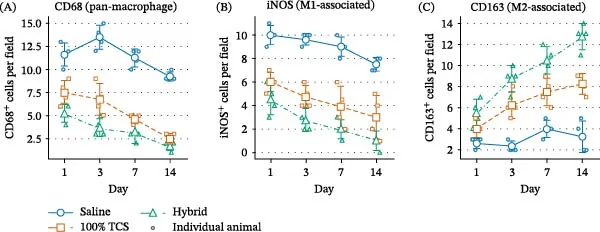
Immunopositive cell counts per high‐power field over 14 days for (A) CD68, (B) iNOS, and (C) CD163. Filled symbols and connecting lines show group means with error bars representing ± 1 SD; open symbols show the individual values of all four animals in each subgroup. Pooled across timepoints, the counts in the hybrid group were lower than the commercial standard for CD68 and iNOS and higher for CD163, with effect estimates and confidence intervals shown in Table 4. The group × timepoint interaction between the hybrid and commercial standards was not significant for any marker.

#### 3.3.1. Pan‐Macrophage Infiltration (CD68)

In the saline group, CD68‐positive cell counts peaked on day 3 (13.50 ± 1.29 cells per field) and remained elevated on day 14 (9.25 ± 0.50 cells per field). Counts declined progressively in both active groups and were consistently lowest in the hybrid group, falling from 5.25 ± 0.96 at day 1 to 1.75 ± 0.50 at day 14, a reduction of ~67%, compared with 7.50 ± 1.29 to 2.50 ± 0.58 for the commercial standard (Figure 8A).

#### 3.3.2. M1‐Associated Marker (iNOS)

The saline group remained iNOS‐predominant throughout the study (10.00 ± 0.82 on day 1 and 7.50 ± 0.58 on day 14). The counts in the hybrid group were the lowest at every time point, declining from 4.50 ± 1.29 on day 1 to 1.00 ± 0.82 on day 14 (~78%), compared with 6.00 ± 0.82–3.00 ± 1.83 for the commercial standard group (Figure 8B).

#### 3.3.3. M2‐Associated Marker (CD163)

CD163‐positive counts were low in all groups during the early phase and increased thereafter in both active groups, most markedly in the hybrid group, which reached 10.50 ± 1.29 at day 7 and 12.75 ± 1.26 at day 14 versus 7.50 ± 1.29 and 8.25 ± 0.96, respectively, for the commercial standard, an increase of ~132% from the day‐1 hybrid value (5.50 ± 1.29) (Figure 8C).

In the aligned rank‐transform factorial analysis, the group × timepoint interaction across all three groups was significant for CD68 (*p* = 0.037) and CD163 (*p* = 0.002) but not for iNOS (*p* = 0.858), reflecting the divergence of the untreated group from that of the treated groups. When restricted to the comparison of primary interest (hybrid versus commercial standard), the interaction was not significant for any marker (CD68, *p* = 0.128; iNOS, *p* = 0.974; CD163, *p* = 0.119), whereas the main effect of the group remained significant for all three (*p* < 0.001). Therefore, the data support a consistent difference in marker levels between the hybrid and commercial standards rather than a demonstrably different rate of change.

## 4. Discussion

The survival of the pulp after deep exposure depends on whether the capping material can nudge acute inflammation toward a proresolving dentinogenic state [14, 35]. In this in vivo comparison, the water‐activated 75:25 TCS/*A. fulica* CaCO_3_ formulation—measured against the commercial calcium‐silicate standard—was associated with lower pan‐macrophage (CD68) and M1‐associated (iNOS) marker counts, higher M2‐associated (CD163) counts, and hepatorenal values markedly lower than those of the commercial standard, with less severe renal histopathology [30, 32].

A mechanically injured pulp is quickly saturated with pattern recognition signaling, and when the inflammatory infiltrate is not brought under control inside a rigid, poorly collateralized chamber, ischemia and necrosis can follow [2, 3]. The saline group illustrated this: CD68‐positive recruitment surged by day 3 and remained elevated on day 14. The counts in the hybrid group were lower than those in either comparator group at every time point, a pattern compatible with a curtailed chemotactic gradient [9, 24]. Because the group × timepoint interaction between the hybrid and commercial standard was not significant for any marker, this should be read as a difference in level throughout the observation period rather than a faster resolution.

Persistent M1‐associated activity and failed barrier closure tend to be correlated. Switched on by damage‐associated molecular patterns and NF‐κB, M1 macrophages generate nitric oxide through iNOS, which, together with reactive oxygen species, can damage DNA and stall pulp stem cell maturation [8, 9, 40]. TCS cement eventually reduces iNOS counts, but its rapid dissolution unloads a large hydroxyl burden (pH >12.5) that can coagulate the superficial tissue on contact with pulp fluid [18]. This early alkaline insult may add fresh damage signals and prolong resolution, an interpretation consistent with, though not proven by, the higher iNOS and CD68 counts seen with the commercial standard through the first 72 h [19, 41].

The addition of biogenic CaCO3 may help buffer this early alkaline stress via the carbonate–bicarbonate equilibrium [25]. We further speculate that calcium ions released from the extract could modulate NF‐κB signaling, a hypothesis requiring direct molecular confirmation [6, 50]. Because the hydration vehicle also differed between groups and neither interfacial pH nor NF‐κB signaling was measured here, these are offered as plausible contributors rather than demonstrated causes; together, they may partly explain the lower iNOS counts observed with the hybrid. Notably, the measured pH of the set hybrid (11–12) does not provide evidence of buffering to the near‐neutral range sometimes proposed for carbonate‐modified cements.

Compositionally, the hybrid took up carbonate groups, which was evident from its higher surface oxygen and carbon content, while the surface calcium fraction remained close to that of the commercial cement at 23.7 wt% [51]. Because EDX is surface‐sensitive, semiquantitative, and derived from a single map‐sum spectrum per material, this observation should not be interpreted as evidence of stable calcium retention or sustained calcium availability, and it is not the increase that the calcium‐richer extract would lead one to expect. Swapping the proprietary calcium‐chloride liquid for deionized water lowered the chlorine content [52], whereas the calcite‐dominant character of biogenic CaCO_3_ may furnish additional nucleation sites during hydration [38, 39]. These findings are consistent with recent in vivo work by Pratama et al. [50], who reported that bioceramics hybridized with biogenic calcium carbonate from *Anadara granosa* shells modulated NF‐κB and TGF‐β1 signaling in a rat model.

Lower pan‐macrophage and M1‐associated counts were accompanied by higher CD163‐associated counts. Within the cramped pulp microenvironment, the accumulation of CD163‐positive macrophages beneath the capping interface is generally regarded as a precondition for healing, tracking with growth factor secretion, and resolution of inflammation [7, 20]. The ~132% increase in CD163‐positive counts in the hybrid group between days 1 and 14 was consistent with a proresolving macrophage marker profile [10, 20]. It is important to be explicit about what this does and does not show: because CD68, iNOS, and CD163 were assessed in independent cross‐sectional samples and without colocalization or multiplex staining, these data cannot establish that individual macrophages changed phenotype, and no claim of phenotypic transition, repolarization, or reparative dominance is made in this study.

The systemic spread of leachable particles from unset dental materials is a well‐recognized concern in the field of toxicology [31]. The slower hydration of the commercial cement may have permitted greater early release of unbound elements, paralleled by ALT and creatinine elevations that persisted until day 14 in that group [53]. In the hybrid group, the lower chlorine content may have limited early leaching; however, the simultaneous change in the hydration vehicle means that calcium carbonate cannot be credited in isolation, and no material‐derived element was measured in the serum or organ tissue. Hepatorenal biochemistry and organ histology in the hybrid group showed mild, predominantly renal changes that were less severe than those with the commercial standard, although creatinine, BUN, ALT, and AST all remained above the operated saline control, a profile more favorable than, but not equivalent to, that of the control [53, 54]. One hepatic finding runs counter to the overall pattern and should not be overlooked: portal inflammation was modestly higher in the hybrid group than in the control group. Given the small subgroup size and low absolute scores, this observation warrants confirmation rather than a mechanistic explanation for the findings.

### 4.1. Limitations

This study had several limitations. The comparison is confounded because the groups differed in terms of carbonate content, hydration vehicle, and powder fraction; therefore, the effects are not attributable to calcium carbonate alone, requiring a factorial design for the study. The effect size was assumed, not pilot‐derived; timepoint subgroups (*n* = 4) were underpowered and reported descriptively; the study was exploratory; and meloxicam rendered early kinetics not entirely material‐driven. Pulpal assessment used three immunohistochemical markers without hematoxylin–eosin grading, staining/sectioning records, or reliability testing; 14 days precluded bridge formation; the surgeon was unblinded; and mechanical exposure lacked the bacterial component of carious pulpitis. The systemic assessment lacked baseline and unoperated controls, and no material‐derived elements were measured. The marginal aggregate hepatic score was not robust for alternative record handling (File S1). Characterization used single FTIR and EDX spectra with unrecorded parameters, no X‐ray diffraction, thermogravimetry, contaminant screening, or particle size analysis, and nonstandardized pH/setting time methods. Figure 6 lacks calibrated scale bars, and the proposed mechanisms are thus hypothetical.

### 4.2. Future Directions

Future studies should employ a fully factorial deconfounding design, longer observation windows with microcomputed tomography and histomorphometry to quantify the calcified barrier, conventional hematoxylin–eosin pulp‐response grading alongside colocalization or multiplex immunostaining to characterize macrophage phenotype directly, direct physicochemical measurement of interfacial pH, ion release, and setting kinetics, ICP‐based elemental analysis of serum and organs, molecular and proteomic analyses (RT‐PCR, proteomics) to test the NF‐κB, STAT1, and STAT6 pathways, and—only after such preclinical validation—early phase clinical evaluation.

## 5. Conclusions

Within the limits of this exploratory in vivo model, a water‐activated hybrid of 75% TCS and 25% *A. fulica* calcium carbonate was compared with the manufacturer‐prepared commercial calcium‐silicate standard and was associated with lower pro‐inflammatory (M1‐associated) and higher proresolving (M2‐associated) macrophage marker counts (lower CD68 and iNOS and higher CD163), lower hepatorenal enzyme and metabolite values, and less severe renal histopathology, although all systemic parameters remained above those of the operated saline control, and portal inflammation was modestly higher in the hybrid group. Because the two formulations differed in more than one respect, these findings represent the net response to the complete formulation rather than the isolated action of calcium carbonate. Furthermore, because they rely on cross‐sectional marker immunoreactivity, they do not demonstrate macrophage phenotype switching, dentinogenesis, or systemic biocompatibility. These results support further preclinical evaluation of this hybrid for vital pulp therapy and motivate factorial, longer‐term, and molecular studies to define the responsible mechanisms.

## Author Contributions

**Endo Sadewa**: conceptualization, methodology, investigation, formal analysis, data curation, writing – original draft. **Nurhayaty Natsir:** supervision, validation, writing – review and editing. **Nurlindah Hamrun**: supervision, writing – review and editing. **Noor Hikmah**: supervision, validation, writing – review and editing. **Nisrina Ekayani Nasrun**: validation, writing – review and editing. **Kezia Rachellea Mustakim**: conceptualization, visualization. **Juni Jekti Nugroho**: supervision, project administration, validation, writing – review and editing.

## Funding

This study did not receive any specific funding.

## Disclosure

All authors have read and approved the final version of the manuscript. The corresponding author, Juni Jekti Nugroho, had full access to all the data in this study and took complete responsibility for the integrity and accuracy of the data analysis.

## Ethics Statement

All surgical procedures, husbandry, and euthanasia performed on the animals in this study complied with the ARRIVE 2.0 guidelines and international standards for the welfare of experimental animals. The study protocol was reviewed and approved by the Institutional Health Research Ethics Committee of the Faculty of Dentistry, Hasanuddin University (Approval Number 347/KEPK FKG‐RSGMP UH/EA/XII/2025). This study was conducted exclusively using an in vivo animal model and did not involve any human participants.

## Consent

The authors have nothing to report.

## Conflicts of Interest

The authors declare no conflicts of interest.

## Supporting Information

Additional supporting information can be found online in the Supporting Information section.

## Supporting information


**Supporting Information** File S1: Complete per‐animal dataset for all 48 animals, comprising immunohistochemical cell counts (CD68, iNOS, and CD163), hepatorenal biochemistry (creatinine, BUN, AST, and ALT), and semiquantitative liver and kidney histopathology scores, together with a record of data provenance (Microsoft Excel file). File S2: Analysis script that reproduces every descriptive and inferential statistic reported in the manuscript from File S1 and reconciles each computed value against the published value (Python file). File S3: Complete Fourier‐transform infrared instrument peak table for the 13 bands detected in the *Achatina fulica* shell‐derived calcium carbonate, with band positions, intensities, baseline limits, areas, and assignments (Microsoft Excel file).

## Data Availability

The complete per‐animal dataset underlying every table and figure in this article (immunohistochemical counts, hepatorenal biochemistry, and semiquantitative liver and kidney histopathology scores for all 48 animals) is provided as File S1, the analysis script that regenerates every reported statistic as File S2, and the complete Fourier‐transform infrared instrument peak table as File S3. No other data were generated or analyzed in this study period.
